# Long-term response to pimitespib in postoperative recurrent gastrointestinal stromal tumors with *PDGFRA* D842V mutation: a case report

**DOI:** 10.1186/s40792-023-01637-4

**Published:** 2023-04-07

**Authors:** Ryugo Teranishi, Tsuyoshi Takahashi, Yukinori Kurokawa, Takuro Saito, Kazuyoshi Yamamoto, Kotaro Yamashita, Koji Tanaka, Tomoki Makino, Kiyokazu Nakajima, Hidetoshi Eguchi, Yuichiro Doki

**Affiliations:** Department of Gastroenterological Surgery, Osaka University Graduate School of Medicine, 2-2-E2 Yamadaoka, Suita, Osaka 565-0871 Japan

**Keywords:** GIST, PDGFRA, exon18 D842V mutation, Pimitespib, Avapritinib, HSP90, Imatinib resistant

## Abstract

**Background:**

Exon 18 D842V, which is a point mutation from aspartic acid to valine at codon 842, is the most frequent mutation in Platelet-Derived Growth Factor Receptor alpha (*PDGFRA*)-mutated gastrointestinal stromal tumor (GIST). In the Japanese GIST guidelines, no standard systematic therapy is available for this type of GIST, which is refractory after recurrence. Recently, pimitespib (PIMI), a novel heat shock protein 90 (HSP90) inhibitor, was approved for the treatment of advanced GIST in a phase III study. This report presents a case of a long-term response to PIMI in GIST with *PDGFRA* D842V mutation.

**Case presentation:**

A 55-year-old woman was diagnosed with primary GIST of the stomach and underwent partial gastrectomy. Eight years after the operation, recurrent GISTs were identified as multiple recurrent peritoneal GISTs in the upper right abdomen and pelvic cavity. We administered tyrosine kinase inhibitors, but they achieved poor effects. After failure of the standard treatment, PIMI was administered and achieved a partial response in the patient. The highest reduction rate was 32.7%. After PIMI failed, we performed multiplex gene panel testing, which revealed the *PDGFRA* D842V mutation.

**Conclusions:**

We report the first case of long-term response to PIMI in *PDGFRA* D842V mutant GIST. Pimitespib may be effective for treating GIST harboring this mutation by inhibiting HSP90.

## Background

Gastrointestinal stromal tumor (GIST) is the most common type of gastrointestinal mesenchymal tumors [1]. Approximately 90% of GIST have a gain of function mutations in *KIT* or Platelet-Derived Growth Factor Receptor alpha (*PDGFRA*) [2, 3]. The activation of KIT or PDGFRA receptor tyrosine kinase plays a crucial role in the proliferation of GIST [4]. Tyrosine kinase inhibitors (TKIs) targeted for GIST, such as imatinib (IM), sunitinib, and regorafenib, have been approved as first-, second-, and third-line therapy, respectively [5, 6].

GIST with PDGFRA mutations account for approximately 10% of all GIST [7]. *PDGFRA*-mutated GIST occurs most frequently in the stomach [8]. Pathological examinations have revealed several characteristic morphological features, such as epithelioid pattern and myxoid stroma [8, 9]. They tend to follow a more indolent clinical course and have a 70% lower risk of 5-year relapse than patients with KIT mutations [10]. *PDGFRA* mutations are found mainly in exons 12 and 18, and rarely in exon 14. The most frequent mutation in exon 18 is D842V, which is a point mutation from aspartic acid to valine at codon 842 and detected in 75% of all PDGFRA-mutated GISTs [8, 11]. This mutation is primarily resistant to type 2 TKIs, such as IM, and has a poor prognosis with a median progression-free survival (PFS) of 2.8 months [3, 12–14]. According to the European Society for Medical Oncology (ESMO) and National Comprehensive Cancer Network (NCCN) guidelines, IM is not recommended for use in GISTs with *PDGFRA* exon18 D842V mutation, as this type of GIST is refractory to treatment [15, 16].

Recently, pimitespib (PIMI), a novel heat shock protein 90 (HSP90) inhibitor, was developed [17, 18]. HSP90 regulates the conformation, function, and activation of several client proteins related to cancer growth, including KIT and PDGFRA [19, 20]. PIMI selectively binds to cytoplasmic HSP90α and HSP90β and inhibits HSP90 enzymatic activity [21]. Inhibition of HSP90 downregulates multiple signaling pathways in tumor cells and leads to anti-carcinogenesis [20, 22]. In Japan, a phase II study of PIMI was conducted in patients with advanced GIST who failed or were intolerant to IM, sunitinib, and regorafenib [23]. PIMI has shown promising results in this refractory population. Subsequently, a phase III (CHAPTER-GIST-301) study was conducted [24], and it was revealed that PIMI significantly improved PFS compared with placebo, with a median PFS of 2.8 months. Based on these results, PIMI received insurance approval in June 2022 for the indication of advanced GIST.

This report presents a case of long-term response to PIMI in GIST with *PDGFRA* D842V mutation.

## Case presentation

**Fig. 1:**
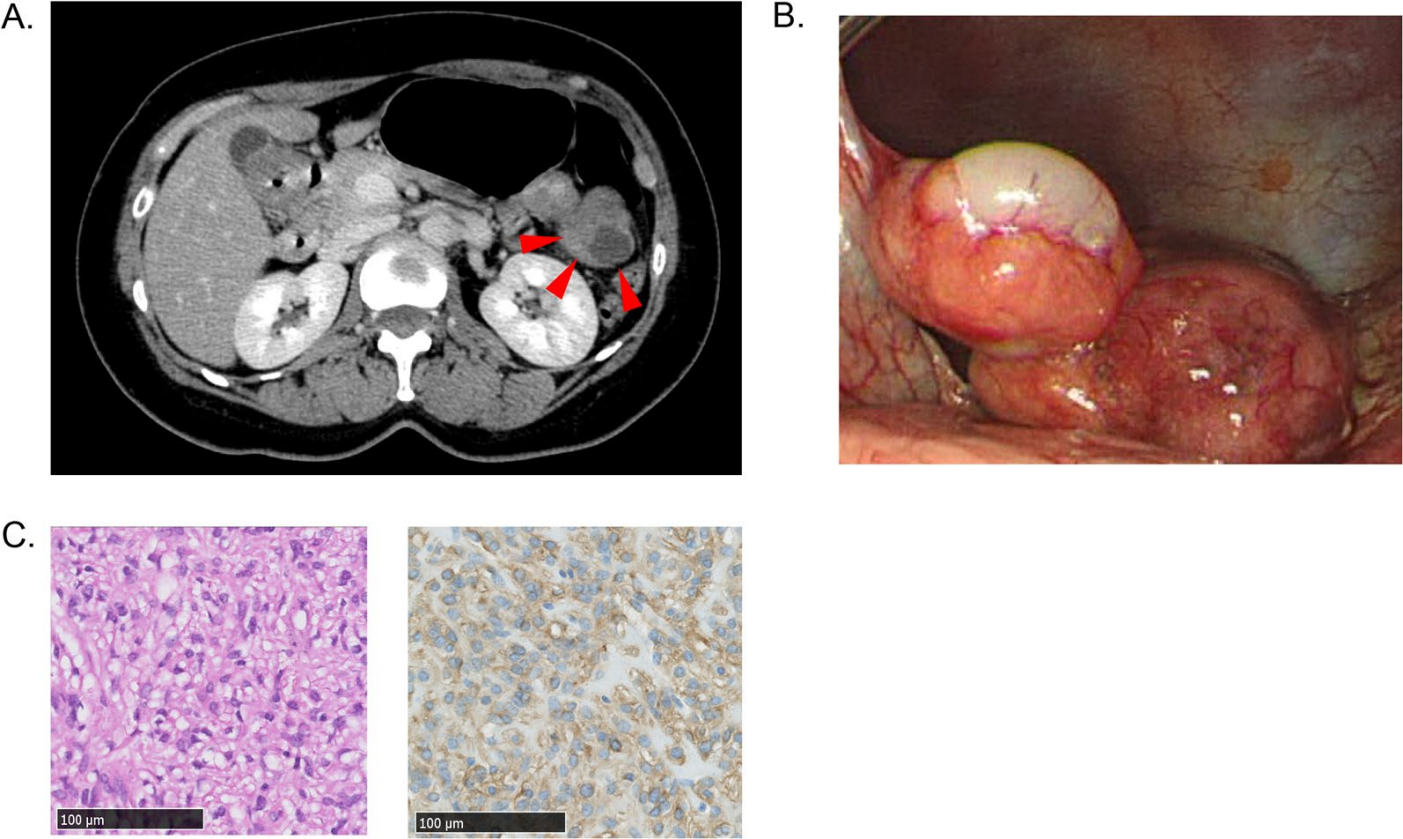
Perioperative patient information. **A** Abdominal computed tomography at pre-operation. **B** Intraoperative findings. **C** Hematoxylin and eosin staining (×400) and immunohistochemical staining for KIT/CD117 (×400). Red arrowheads: primary GIST of the stomach

A 55-year-old woman was diagnosed with primary GIST of the stomach and underwent partial gastrectomy (Fig. 1A, B). The tumor stained positively for CD117 (KIT) and was composed of mixed epithelioid/spindle cells with five mitoses/50 high-power fields (Fig. 1C). This tumor is classified as intermediate-risk based on the modified Fletcher risk classification. Based on the gene analysis and the later multiplex gene panel testing, we confirmed that this tumor had a *PDGFRA* D842V mutation.

**Fig. 2:**
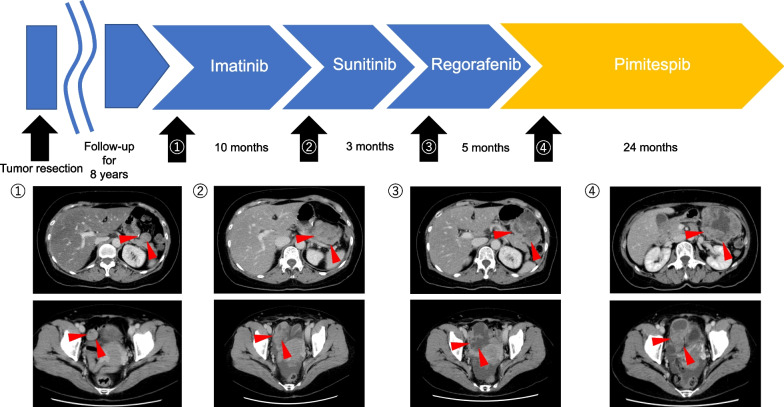
Clinical course of treatments. Red arrowheads: recurrent peritoneal GISTs in the upper right abdomen and pelvic cavity

The patient was followed-up without adjuvant chemotherapy. Eight years after the operation, recurrent GISTs were identified as multiple recurrent peritoneal GISTs in the upper right abdomen and pelvic cavity. According to GIST guidelines, IM was administered for 10 months, sunitinib for 3 months, and regorafenib for 5 months (Fig. 2). However, these agents achieved poor effects.

**Fig. 3:**
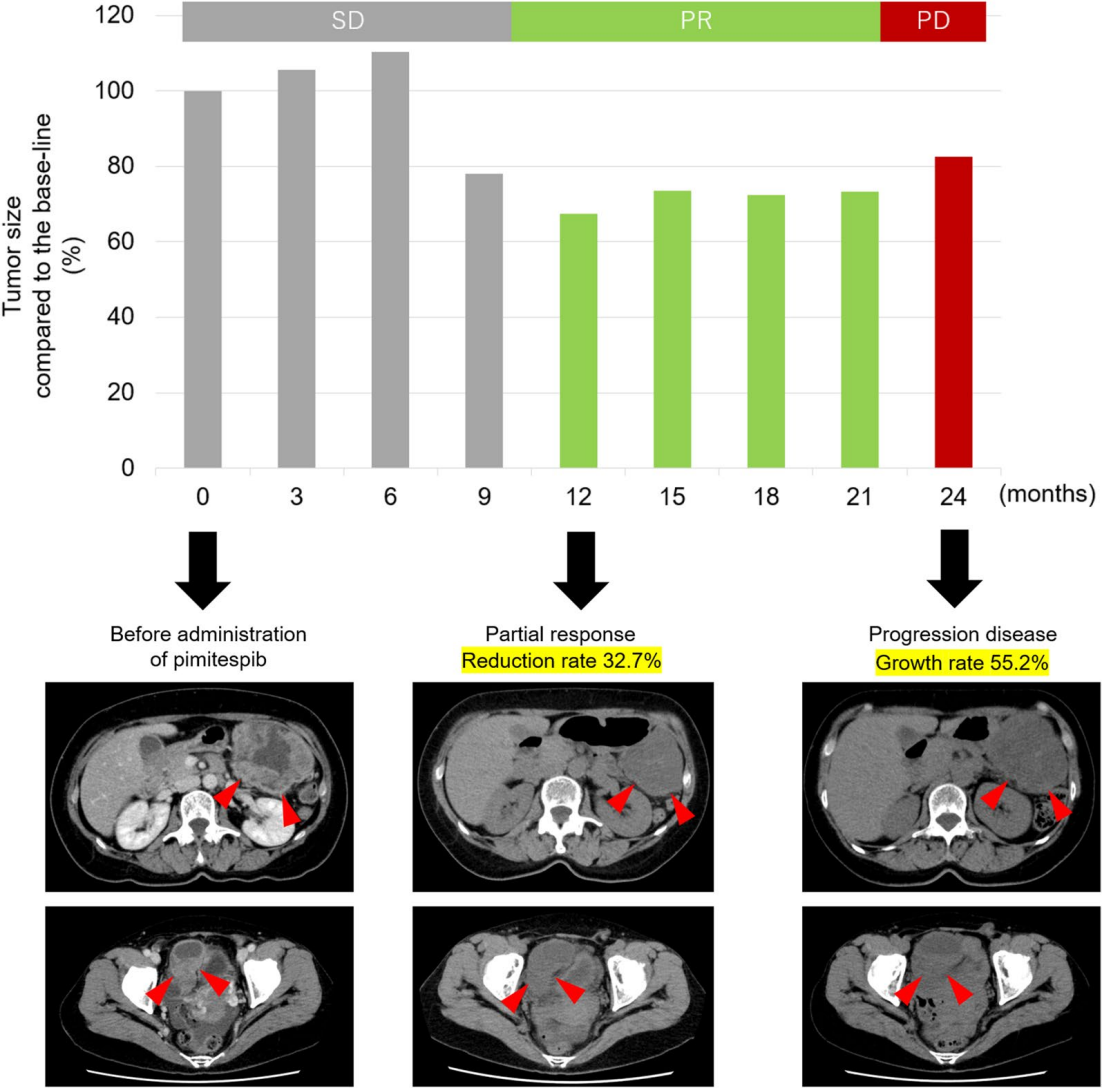
Detail of effect during treatment with PIMI. Tumor effects are based on response evaluation criteria in solid tumors ver 1.1. *PIMI* pimitespib. Red arrowheads: recurrent peritoneal GISTs in the upper right abdomen and pelvic cavity

Next, we introduced her to the clinical study (CHAPTER-GIST-301) [24], and she agreed to enroll. The patients were assigned to the PIMI group. She received oral PIMI 160 mg/day under fasting conditions for 5 consecutive days, followed by a 2-day rest in a 21-day cycle (Fig. 3). She experienced only tolerable diarrhea (Common Terminology Criteria for Adverse Events, Grade 2). For the first 8 months after the initiation of PIMI administration, the tumor size remained stable, and the effect of PIMI was slight. However, at the ninth month of administration, the patient achieved a partial response (Fig. 3). The highest reduction rate was 32.7%. Twenty-four months after PIMI administration, abdominal computed tomography detected tumor regrowth. Therefore, we determined that the patient had progressive disease. After PIMI failure, we performed the cancer multi-gene panel testing. However, there was no gene mutation related to the clinical trials. Furthermore, since we couldn’t expect the effect of TKIs for *PDGFRA* exon 18 D842V mutant GIST, re-challenge with TKIs was not indicated. We finally decided the treatment policy for best supportive care.

## Discussion

In this case, recurrence occurred long after curative surgery. Subsequent treatment with standard TKIs was ineffective. This clinical course of slow growth and resistance to TKIs is typical for *PDGFRA* D842V mutant GIST [3, 10, 12–14, 25, 26]. PIMI is an HSP90 inhibitor, and its mechanism of action differs from that of TKIs. As a result, PIMI successfully controlled tumor activity for a long period. There are no previous reports on the response of *PDGFRA* D842V mutant GIST to an HSP90 inhibitor. PIMI is expected to be effective in GIST patients with the *PDGFRA* D842V mutation.

In a phase III study, the incidence of treatment-related adverse event (AE) leading to permanent discontinuation was only 5.2% [24]. Furthermore, eye disorders occurred in only 3.4% of the cases and were much fewer than those in previous reports of other HSP90 inhibitors [27, 28]. Therefore, PIMI is accepted to have favorable safety. Nevertheless, 25.9% of all patients experienced grade ≥ 3 AE, and dose interruptions and reductions due to AE were common (58.6% and 34.5%, respectively) in a phase III study [24]. In this case, PIMI was administered continuously for 2 years, and the PFS was much longer than that in the phase III study. Dose interruption and reduction may lead to tumor progression. The patient experienced only grade 2 diarrhea, and we were able to maintain the PIMI dosage. The good tolerability of PIMI may have contributed to this benefit.

IM can bind only to the inactive conformation of tyrosine kinase receptors [29, 30]. In the *PDGFRA* exon 18 D842V mutation, the kinase activation loop is distorted, resulting in a strong tilting toward a protein conformation that favors activation and is generally believed to lead to primary IM resistance [7, 31]. Therefore, in the NCCN and ESMO guidelines, IM and other TKIs are not indicated for *PDGFRA* exon 18 D842V mutant GIST [15, 16]. There is no standard therapy available for this GIST molecular subtype, and surgical resection is preferred. Recently, avapritinib, a type 1 TKI that inhibits potent and highly selective PDGFRA mutant kinases, has been developed [13]. A phase I NAVIGATOR trial that evaluated the safety and antitumor activity of avapritinib in patients with *PDGFRA* D842V mutant GIST was conducted [32]. The overall response rate with avapritinib was 91%, and the median PFS was 34.0 months. These results were remarkable in a GIST molecular subtype known to be refractory to other TKIs. Based on this trial, avapritinib was approved by the Food and Drug Administration for patients with advanced GIST harboring *PDGFRA* exon 18 mutations, including the D842V mutation [32, 33]. In the current NCCN and ESMO guidelines, avapritinib is indicated as the first-line treatment for GIST with *PDGFRA* D842V mutation [15, 16]. However, avapritinib has not been approved in East Asia, including Japan and South Korea, and this approval lag is a serious issue. In this case, PIMI was effective against this mutation through a mechanism different from that of avapritinib, that is, by inhibiting HSP90. PIMI may be a promising treatment for *PDGFRA* D842V mutant GIST, in addition to avapritinib.

In a phase III study, the best response was stable disease (62.1%), with no complete response or partial response [24]. This is the first case of GIST with a partial response to PIMI. However, the detailed mechanism by which the good response was observed in this case is still unknown. To predict the drug effect by the patient’s factors including mutation type in detail, further analysis is needed.

## Conclusion

We encountered a case of *PDGFRA* D842V mutant GIST with a long-term response to PIMI. PIMI may be effective for treating GIST harboring this mutation by inhibiting HSP90.

## Data Availability

The data that support the findings of this study are available upon request from the corresponding author, Tsuyoshi Takahashi. The data are not publicly available because they contain information that can compromise the privacy of the research participants.

## Abbreviations

AE: Adverse event
D842V: Aspartic acid to valine on codon 842
ESMO: European Society for Medical Oncology
GIST: Gastrointestinal stromal tumor
HSP90: Heat shock protein 90
IM: Imatinib
NCCN: National Comprehensive Cancer Network
PDGFRA: Platelet-Derived Growth Factor Receptor alpha
PIMI: Pimitespib
PFS: Progression-free survival
TKIs: Tyrosine kinase inhibitors
